# In vitro antimicrobial and antibiofilm screening of eighteen Iranian medicinal plants

**DOI:** 10.1186/s12906-024-04437-x

**Published:** 2024-03-28

**Authors:** Maryam Hamidi, Ali Mohaghegh Toosi, Behjat Javadi, Javad Asili, Vahid Soheili, Abolfazl Shakeri

**Affiliations:** 1 Department of Pharmacognosy, School of Pharmacy, Mashhad University of Medical Sciences, Mashhad, Iran; 2 Department of Pharmaceutical Control, School of Pharmacy, Mashhad University of Medical Sciences, Mashhad, Iran; 3 Department of Traditional Pharmacy, School of Pharmacy, Mashhad University of Medical Sciences, Mashhad, Iran

**Keywords:** Screening, Natural products, Antimicrobial, Antibiofilm, *Hypericum scabrum*, *Hymenocrater calycinus*

## Abstract

**Background:**

Natural products are one of the best candidates for controlling drug-resistant pathogens, the advantages of which include low production costs and low side effects. In this study, as potential antimicrobials, the anti-bacterial and antibiofilm activities of several Iranian native medicinal plants were screened.

**Methods:**

The antibacterial/antifungal and anti-biofilm activities of 18 medicinal plants including *Reseda lutea* L*.*, *Nepeta sintenisii* Bunge*.*, *Stachys turcomanica* Trautv., *Stachys lavandulifolia* Vahl, *Diarthron antoninae* (Pobed.) Kit Tan., *Ziziphora clinopodioides* Lam., *Euphorbia kopetdaghi* Prokh, *Euphorbia serpens* Kunth., *Hymenocrater calycinus* Benth., *Scutellaria pinnatifida* A.Ham*.*, *Viola tricolor* L., *Hypericum helianthemoides* (Spach) Boiss., *Hypericum scabrum* L., *Convolvulus lineatus* L., *Scabiosa rotata* M.Bieb Greuter & Burdet, *Delphinium semibarbatum* Bien. Ex Boiss., *Glycyrrhiza triphylla* Fisch. & C.A.Mey., and *Ziziphus jujuba* Mill., against two Gram-positive bacteria, *Staphylococcus aureus*, *Bacillus cereus*, as well as two Gram-negative bacteria, *Pseudomonas aeruginosa*, *Escherichia coli;* and *Candida albicans* as a fungal strain, were evaluated. The minimum inhibitory concentration (MIC) and minimum bactericidal/fungicidal concentration (MBC/MFC) values of the extracts against tested microorganisms were reported and we investigated their effect on the biofilm inhibition of *Pseudomonas aeruginosa* PAO1, *Staphylococcus epidermis*, *Staphylococcus aureus* and *Streptococcus mutans*. In addition, the effect of the extracts on the eradication of the biofilms of these bacteria was evaluated.

**Results:**

In this study, *H. scabrum* was found to exhibit potentially significant activity against Gram-positive bacteria with the MIC range of 6.25–25 µg/mL. This extract also showed a significant effect on inhibiting the biofilm of *S. aureus,* S. *mutans*, and *S. epidermidis* and eradicating the biofilm of *S. epidermidis* DSMZ 3270. In addition, *Hymenocrater calycinus* root extract had moderate antibacterial activity against *B. cereus* with the MIC and MBC 62.5 µg/mL, respectively.

**Conclusions:**

The results of this study showed that the root extracts of two plants, *Hypericum scabrum* and *Hymenocrater calycinus*, had antimicrobial and anti-biofilm effects. Based on the observed anti-biofilm effects, these two plants may be considered in future studies to find responsible antimicrobial compounds.

## Introduction

Drug-resistant bacteria are currently a major worldwide health concern, as they are not responding well to conventional treatments [1]. By finding novel antibiotics and chemically altering existing antimicrobial medications, it has been sought to overcome bacterial resistance to antimicrobial drugs [2]. It has been estimated that biofilms are responsible for approximately 75% of bacterial infections [3]. Depending on how genetic products function and what type of drug molecule, there are several kinds of drug resistance. Bacteria develop secondary resistance to substances when they are exposed to them repeatedly and several factors can contribute to this development [4].

Biofilms are a major concern for the food and medical industries as they have been linked to numerous hospital infections and food spoiling problems [5]. Biofilm cells are capable of irreversibly adhering to surfaces, living tissues, and indwelling medical devices such as catheters, valves, prostheses, etc. [3]. Bacterial cells embedded in a biofilm exhibit increased resistance to antimicrobial agents and other environmental stresses compared to free-floating cells. Finding new agents to combat biofilm formation and providing a substitute for routine medication would be of great importance [6]. The use of plant-based medicines is an effective alternative that may help minimize the negative effects of conventional antibiotic therapy. Additionally, plant-based formulations would enhance the amount of anti-infection chemicals available and may function in combination with existing treatments to reduce infections [7]. Medicinal plants can also be used as antibacterial and antifungal agents. Several studies have reported the anti-microbial and anti-biofilm activity of various medicinal plants [8], which highlights the need for screening herbal extracts and natural compounds to discover novel antimicrobial agents. Iran, as a vast country with diverse climatic features in Asia, has approximately 8167 identified plant species, among which are almost 1900 endemic [9, 10].

In the course of our screening study to discover new antimicrobial compounds from Iranian medicinal plants [11], we have screened 18 plants which are based on their traditional use. *R. lutea*, *Z. clinopodioides*., *E. serpens*, *H. calycinus*, and *C. lineatus* are used as antimicrobials by local people. Additionally, the antimicrobial activity of some other plant species in this study has demonstrated, *N. sintenisii* [12], *S. lavandulifolia* [13],* S. pinnatifida* [14], *V. tricolor* L. [15], *H. helianthemoides* [16], *H. scabrum* [17], *G. triphylla* [18], and *Z. jujuba* [19].

It was reported that using a binary solvent mixture with one polar and a non-polar component (MeOH- CH_2_Cl_2_, 1:1) could extract diverse bioactive compounds from plant materials [20, 21]. Therefore, in our study, the preparation of the extracts was performed using this combination.

## Materials and methods

Tryptic soy agar (TSA) and Sabouraud dextrose broth (SDB) were supplied from HiMedia, India. Glucose and crystal violet, dimethyl sulfoxide (DMSO), and 2,3,5-triphenyl tetrazolium chloride (TTC) were provided from Merck, Germany. Mueller Hinton broth (MHB) was purchased from Liofilchem, Italy, and cloxacillin was obtained from Farabi, Iran. All solvents used were of analytical grade and provided by Dr. Mojallali Industrial Chemical Complex Co, Iran.

### Extract preparation

All the plant species were collected from various locations and identified by Ms. Souzani and have been deposited at the Herbarium of the School of Pharmacy, Mashhad University of Medical Sciences, Iran. The dried plant parts were powdered and extracted with dichloromethane/methanol (1:1) using the maceration method at 20–22 ^°^C for 24 h (3 times). The obtained extracts were then filtered and dried under vacuum by rotary evaporator. The extracts were kept at -20 °C until being used.

#### Bacterial strains

To screen the antimicrobial activity of the extracts, five microorganisms were used as representative bacteria and fungi including *Staphylococcus aureus* (ATCC 25293) and *Bacillus cereus* (PTCC 1023), as Gram-positive bacteria, as well as *Pseudomonas aeruginosa* (PTCC 1074), and *Escherichia coli* (PTCC 1330) as Gram-negative bacteria; and *Candida albicans* (PTCC 5027) as fungal strain. All microorganisms were purchased from the Persian Type Culture Collection (PTCC). Biofilm-producer strains of bacteria including *P. aeruginosa* PAO1 (Nottingham wild type), *Streptococcus mutans* (ATCC 35668), and *Staphylococcus epidermidis* (DSMZ 3270) were also used in further assessments. While the bacterial strains were cultured overnight at 37 °C in Tryptic soy agar (TSA), the fungi were cultured for 3 days at 25 °C in sabouraud dextrose agar (SDA), according to the United States Pharmacopeia (USP).

#### Minimum inhibitory concentration (MIC) and minimum bactericidal/fungicidal concentration (MBC/MFC)

The procedure was performed in 96-well plates according to the microdilution method of CLSI standard. The extracts were dissolved in DMSO at a concentration of 25 mg/200–500 μL and subsequently, the volume reached 5 mL. This reference concentration (5 mg/mL) was used to prepare the first concentration of the assessment (1 mg/mL). The other concentrations (500–0.97 µg/mL) were prepared by the serially twofold dilution method. In the next step, 180 μL of various concentrations of the extracts were inoculated into the wells. Subsequently, 20 μL of bacterial/fungal suspension (10^6^ $\frac{CFU}{ml}$) was added to each well, separately. To evaluate the accuracy of the test, three wells were filled with MHB containing 2.5% DMSO as a negative control (sterility control), and three wells were specified for each microorganism to check its ability to grow in the culture medium (positive control). The plates were incubated at 37 °C for 16 h for bacterial strains and 48 h at 25 °C for the fungus. Finally, 20 μL TTC (5 mg/mL) was added to each well as a coloring indicator and incubated for another 1 h for bacteria and 24 h for the fungus [3]. Each test was done in triple replication. The MIC was considered as the lowest concentration of an extract that inhibited visible growth (absence of changing in color to red).

The MBC/MFC was determined according to the method explained by Sánchez et al. [3] with slight modifications. Briefly, 10 µl from the wells with no color changing was inoculated on the surface of TSA and incubated at 35 °C for 24 h and 25 °C for 72 h for bacteria and fungi, respectively. After incubation, the formation of colonies was investigated. The MBC was considered the lowest concentration with no microbial growth.

#### Assessment of anti-biofilm activity

The anti-biofilm activities of the extracts were investigated in two steps, before biofilm formation (inhibiting biofilm formation) and after biofilm formation (eradication of biofilm structures). The extract concentration was applied at half of their MICs to avoid growth inhibition. Briefly, 180 µL of glucose-enriched MHB culture was mixed with the extracts to reach $\frac{1}{2}$ MIC. Subsequently, 20 µL of the cell suspension, containing 1 × 10^6^ CFU/mL of *Pseudomonas aeruginosa* PAO1, *Staphylococcus epidermis*, *Staphylococcus aureus* and *Streptococcus mutans* were added to each well, separately. In the control groups, four wells were dedicated to the formation of biofilms as the positive control and the negative control is the sterile culture medium to ensure the sterility of the culture medium during the experiment. The plates were incubated at 37°C for 48 h for mature biofilm formation. However, the culture media was refreshed every 12 h. Biofilms constructed at 96-well bottom-flat plates were washed three times with sterile 0.9% NaCl solution to discard non-adhered bacteria and allowed to dry. The total biomass was measured by crystal violet staining (0.03% w/v). Sterile normal saline was used to remove extra crystal violet, and ethanol 95% (200 μl) was employed to extract the bound crystal violet [22]. The experiments were done in triplicate for each extract. Medium with and without microorganisms in the absence of extracts were used as positive and negative controls, respectively. To evaluate the eradication of biofilm, first, 180 µl MHB enriched with glucose was added to the wells. Then, 20 µl of 1 × 10^6^ CFU/mL from microbial suspension of *Staphylococcus epidermis* was added to each well. The plates were incubated for 48 h at 37°C. To investigate the effect of the extract on the eradication of immature and mature biofilm, it was operated in two ways. In the first method, after 24 h, the previous culture medium was replaced with a new nutrient culture medium. Then, after 12 h, the medium was replaced with MHB containing the extract with a concentration equal to $\frac{1}{2}$ MIC. In the second method, after 24 h, we replaced the previous culture medium with MHB containing an extract with a concentration of $\frac{1}{2}$ MIC. This process was repeated 12 h later. The biofilm staining process was done as well as previously. The optical density of each well stained with crystal violet is measured at 590 nm as a biofilm formation indicator [23].

#### Statistical analysis

Statistical analysis was done using SPSS software version 22. One-way analysis of variance (ANOVA) was used to compare the results. A comparison of the means was performed using a Duncan test at a 95% confidence level (*P* < 0.05).

## Results

### Antimicrobial activities

In this study, the antibacterial, antifungal, and anti-biofilm activity of 18 medicinal plants from 11 various families were assayed. Table 1 presents the MIC and MBC/MFC values of the extracts against tested microorganisms. In our experiment, 16 extracts with MIC > 1000 µg/mL could not show their antimicrobial activities. The root extract of *Hymenocrater calycinus* was not effective against Gram-negative bacteria at the tested concentrations but inhibited the growth of *C. albicans* and, at lower concentrations, Gram-positive bacteria. The extracts from roots and aerial parts of *H. scabrum* possess higher antimicrobial effects against Gram-positive bacteria, especially *S. aureus,* and with lower concentrations, it inhibited *C. albicans*. Extracts prepared from the root of *H. scabrum* demonstrated strong growth inhibitory and bactericidal effects against *S. aureus, and S. mutans* with MIC and MBC of 6.25 and 12.5 µg/mL, respectively. In addition, *H. calycinus* root extract showed potent antibacterial activities against *B. cereus* with both MIC and MBC of 62.5 µg/mL. The extracts from other plant species did not show potent antibacterial and antifungal properties. Therefore, only the roots of *H. scabrum* and *H. calycinus* were chosen for further anti-biofilm investigations.

**Table 1:** Minimum inhibitory concentration (MIC) and minimum bactericidal/fungacidal concentration (MBC/MFC) obtained against microorganisms treated with different extracts

**Species**	*P. aeruginosa*		*S. aureus*		*B. cereus*		*C. albicans*		*E. coli*		*S.mutans*	
	***MIC/MBC (µg/mL)***		***MIC/MBC (µg/mL)***		***MIC/MBC (µg/mL)***		***MIC/MFC (µg/mL)***		***MIC/MBC (µg/mL)***		***MIC/MBC (µg/mL)***	
**Reseda lutea L. (aerial parts)**	*N.A	N.A	N.A	N.A	N.A	N.A	N.A	N.A	N.A	N.A	N.A	**N.A**
**N. sintenisii (aerial parts)**	N.A	N.A	N.A	N.A	N.A	N.A	**250**	N.A	N.A	N.A	N.A	**N.A**
**S. turcomanica (aerial parts)**	N.A	N.A	N.A	N.A	N.A	N.A	**250**	N.A	N.A	N.A	N.A	**N.A**
**S. turcomanica (root)**	N.A	N.A	N.A	N.A	N.A	N.A	N.A	N.A	N.A	N.A	N.A	**N.A**
**S. antoninae (aerial parts)**	N.A	N.A	N.A	N.A	N.A	N.A	**250**	N.A	N.A	N.A	N.A	**N.A**
**S. antoninae (root)**	N.A	N.A	N.A	N.A	**500**	**500**	N.A	N.A	N.A	N.A	N.A	**N.A**
**Z. clinopodioides (root)**	N.A	N.A	N.A	N.A	N.A	N.A	N.A	N.A	N.A	N.A	N.A	**N.A**
**E. kopetdaghi (root)**	N.A	N.A	N.A	N.A	N.A	N.A	N.A	N.A	N.A	N.A	N.A	**N.A**
**H. calycinus (aerial parts)**	N.A	N.A	N.A	N.A	N.A	N.A	N.A	N.A	N.A	N.A	N.A	**N.A**
**H. calycinus (root)**	N.A	N.A	**250**	**250**	**62.5**	**62.5**	**500**	N.A	N.A	N.A	N.A	**N.A**
**S. pinnatifida (aerial parts)**	N.A	N.A	N.A	N.A	N.A	N.A	N.A	N.A	N.A	N.A	N.A	**N.A**
**V. tricolor (aerial parts)**	N.A	N.A	N.A	N.A	N.A	N.A	N.A	N.A	N.A	N.A	N.A	**N.A**
**H.helianthemoides (aerial parts)**	N.A	N.A	N.A	N.A	**62.5**	**62.5**	N.A	N.A	N.A	N.A	N.A	**N.A**
***C. lineatus*** ***(aerial parts)***	N.A	N.A	N.A	N.A	N.A	N.A	N.A	N.A	N.A	N.A	N.A	**N.A**
***C. lineatus*** ***(roots)***	N.A	N.A	N.A	N.A	N.A	N.A	N.A	N.A	N.A	N.A	N.A	**N.A**
**S. rotate (aerial parts)**	N.A	N.A	N.A	N.A	N.A	N.A	N.A	N.A	N.A	N.A	N.A	**N.A**
**S. lavandolifolia (root)**	N.A	N.A	N.A	N.A	**500**	**500**	**500**	N.A	N.A	N.A	N.A	**N.A**
**D. semibarbatum (aerial parts)**	N.A	N.A	N.A	N.A	N.A	N.A	N.A	N.A	N.A	N.A	N.A	**N.A**
**H. scabrum (root)**	**300**	N.A	**6.25**	**12.5**	**25**	**50**	**50**	** > 50**	** > 50**	** > 50**	**6.25**	**12.5**
**G. triphylla (root)**	N.A	N.A	N.A	N.A	N.A	N.A	N.A	N.A	N.A	N.A	N.A	N.A
**E. serpens (aerial parts)**	N.A	N.A	N.A	N.A	N.A	N.A	N.A	N.A	N.A	N.A	N.A	N.A
**Z. jujuba (root)**	N.A	N.A	N.A	N.A	N.A	N.A	N.A	N.A	N.A	N.A	N.A	N.A

^*^N. A Not Active (> 1000)

### Biofilm formation and eradication

**Fig. 1:**
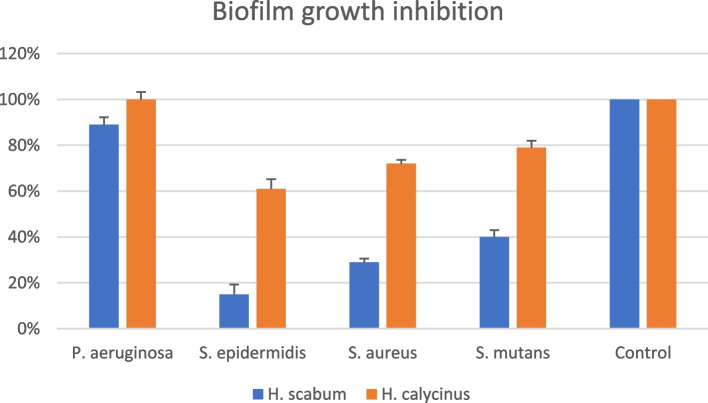
The ratio of biofilm growth in the effect of the *H. scabrum* and *H. calycinus*

**Fig. 2:**
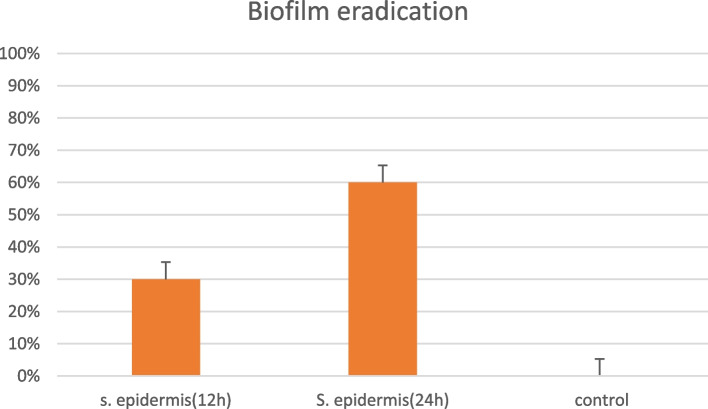
The effect *H. scabrum* on biofilm eradication of *S. epidermidis*

The ability of *H. scabrum* and *H. calycinus* against biofilm formation was assessed against biofilm producer strains, *P. aeruginosa* PAO1, *S. epidermidis*, *S. aureus*, and *S. mutans*. by calculating the optical density ratio (ODr). The results indicated that: (I) both extracts did not show anti-biofilm activity against *P. aeruginosa* in the level of biofilm formation. However, they were able to significantly inhibit the biofilm formation of Gram-positive bacteria (*S. epidermidis* (*P* < 0.05), *S. aureus* (*P* < 0.05), and *S. mutans* (*P* < 0.05), compared to the positive control (Table 2 and Fig. 1). (II) *H. scabrum* showed a significant biofilm eradication (*P* < 0.05) effect against immature and mature-*S. epidermidis* biofilm (Table 3 and Fig. 2).

**Table 2:** Effects of the roots of *H. scabrum* and *H. calycinus* against biofilm formation of *P. aeruginosa*, *S. epidermidis*, *Staphylococcus aureus*, and *Streptococcus mutans*

Microorganisms	*P. aeruginosa*	*S. epidermidis*	*S. aureus*	*S. mutans*
*H. scabrum*	11%	85%	71%	60%
*H. calycinus*	-	39%	28%	21%

**Table 3:** The Effect *H. scabrum* against biofilm eradication of *S. epidermidis*

**Microorganisms**	*S. epidermidis*	*S.epidermidis*
**Tested samples**	***12 h exposure***	***24 h exposure***
*H. scabrum*	30%	*60%*

## Discussion

The present study is an effort to expand our knowledge of the antimicrobial and anti-biofilm effects of some Iranian medicinal plants against bacterial and fungal pathogens. The nature of the organic solvent used for extraction plays an important role in isolating the optimum amounts of various secondary metabolites from the tested plants. In our study, the dichloromethane/methanol (1:1) mixture was speculated to be the best solvent mixture with the ability to extract responsible bioactive metabolites with various polarities. In this study, significant antimicrobial activity against Gram-positive bacteria especially *S. aureus, S. mutans,* and *S. epidermis* was observed in the various parts of *H. scabrum.* Many *Hypericum* species, most notably *H. perforatum*, and their extracts have been shown to possess a range of therapeutic qualities, including anti-inflammatory, antiviral, antimicrobial, antioxidant, antitumoral, and wound-healing effects [24]. The antimicrobial activity of *H. scabrum* previously was reported by several authors [25–27]. For example, Ergin et al. reported that MeOH, n-hexane, and dichloromethane extracts from *H. scabrum* root were effective against *S. aureus* (MIC = 312.5–625 µg/mL) [28]. Also, our present observations are in agreement with another previous study by Keser et al., [29] who reported the potent antimicrobial activity of *H. scabrum* against *E. coli, Proteus vulgaris, P. aeruginosa, Listeria monocytogenes, Klebsielle pneumonia* and *Bacillus subtilis*. It was reported that *H. scabrum* contains a variety of secondary metabolites such as flavonoids [30], xanthones [31], and especially phloroglucinols [32, 33]. It was found that lipophilic fractions of *Hypericum* species contain phloroglucinol derivatives, which have been demonstrated to have antifungal and antibacterial properties against pathogens like *S. aureus, B. cereus, B. subtilis, and Nocardia gardenen* [34]*.* Such compounds could be the cause of the antimicrobial activity, even at relatively low quantities [34]. Furthermore, phloroglucinol and its derivatives are frequently used to treat microbial diseases caused by bacteria, fungi, and viruses [35]. Phytochemical analysis of ethyl acetate extract of the aerial part of *H. scabrum* led to the isolation of eight flavonoids. They have shown moderate to no antimicrobial activity against *S. aureus, E. coli,* and *C. albicans* [36]. Thus, phloroglucinols might be responsible for the potent antimicrobial activity of *H. scabrum.* In our study, also, *H. calycinus* root extract showed good antibacterial activities against *B. cereus* (MIC = 62.5µg/ml). The primary component of *H. calycinus* extract, rosmarinic acid, demonstrated antifungal properties against *C. albicans* [37]. For rosmarinic acid, a wide range of biological actions have been noted. Astringent, anti-oxidative, anti-inflammatory, anti-mutagen, antibacterial, and anti-viral are the major effects [37]. Biofilms are the primary etiological factors in 80% of Human microbial infections. Moreover, biofilms constructed on surgical implants and tissue surfaces are an important threat to human health. Biofilm infections are extremely difficult to treat with common antibiotics, resulting in chronic infections and non-healing wounds. As a result, it has been necessary to develop new antibacterial agents that are effective against biofilm [38]. For instance, New biofilm-inhibiting substances are urgently needed to combat refractory infections like methicillin-resistant *S. aureus* (MRSA) and vancomycin-methicillin-resistant *S. aureus* because biofilms play crucial roles in antibiotic resistance [39]. In our study, the extract of *H. scabrum* demonstrated significant biofilm inhibition against *S. epidermidis* and *S. aureus* and the extract could eradicate *S. epidermidis* at 60% after 24h*.* Interestingly*,* the extracts outperformed common antibiotics (streptomycin sulfate and nystatin) concerning their antimicrobial effects. Pereira et all., found that *Hypericum brasiliense* extract inhibited and eradicated *S. aureus HU25* planktonic cells and their biofilm formation [40]. The anti-biofilm activity of *H. scabrum* could be related to the higher proportion of polyphenols and flavonoids including catechin, quercetin and vanillic acid in the plant, which have been found to possess anti-biofilm activities [41, 42]. The mechanism underlying the anti-biofilm properties of the plant might involve decreasing polysaccharide intercellular adhesion production and altering the composition of exopolysaccharides in *S. epidermidis* [42]. Taken together and based on our current findings, it is possible to employ *H. scabrum* as a source of effective antimicrobial substances to combat infections brought on by vulnerable organisms.

## Conclusions

Our findings established that among 18 Iranian medicinal plants, both *H. scabrum* and *H. calycinus* root extracts had well-potent antimicrobial activities against some tested pathogens. The root of *H. scabrum* extract could inhibit biofilm formation and eradicate the pre-formed biofilm of *S. epidermidis*. However, the clinical application of such anti-microbial compounds needs further both in vitro and in vivo investigations.

## Data Availability

The data supported during the present research are available from the corresponding author upon reasonable request.

## Abbreviations

MIC: Minimum Inhibitory Concentration
MBC/MFC: Minimum Bactericidal/Fungicidal Concentration
TSA: Tryptic Soy Agar
SDB: Sabouraud Dextrose Broth
DMSO: Dimethyl Sulfoxide
TTC: 2,3,5-Triphenyl Tetrazolium Chloride
MHB: Mueller Hinton Broth
PTCC: Persian Type Culture Collection
ATCC: American Type Culture Collection
ODr: Optical Density Ratio
MRSA: Methicillin-resistant *S. aureus*
